# Platelet-rich plasma in non-operative management of mild to moderate carpal tunnel syndrome – A systematic review & meta-analysis of short-term outcomes

**DOI:** 10.1016/j.jor.2021.05.004

**Published:** 2021-05-07

**Authors:** Martin S. Davey, Matthew G. Davey, Eoghan T. Hurley, J. Tristan Cassidy, Hannan Mullett, Niall M. McInerney, John G. Galbraith

**Affiliations:** a Department of Trauma & Orthopaedics, Galway University Hospital, Galway, Ireland; b Royal College of Surgeons in Ireland, Dublin, Ireland; c Department of Orthopaedic Surgery, Sports Surgery Clinic, Dublin, Ireland; d National University of Ireland Galway, Galway, Ireland; e Department of Plastics, Aesthetic & Reconstructive Surgery, Galway University Hospital, Galway, Ireland

**Keywords:** PRP, Platelet rich plasma, CTS, Carpal tunnel syndrome, Median neuropathy

## Abstract

**Background:**

To perform an updated systematic review with meta-analysis on trials focusing on patient-reported outcome measures (PROMs), nerve conduction studies (NCS) result and cross sectional area (CSA) measurements of those who underwent PRP injection for mild to moderate CTS, versus a control.

**Conclusion:**

This study indicates that there may be a potential role for the use of PRP in the non-operative management of mild to moderate CTS results in improvements in pain scores, functional outcomes as well as CSA measurements of the MN at short-term follow-up. However, PRP does not result in improvements in NCS.

**Level of evidence:**

II; Systematic Review & Meta-Analysis of Prospective Trials;

## 1 Introduction

Carpal tunnel syndrome (CTS) is the most common mono-neuropathy accounting for approximately 90% of peripheral entrapment neuropathies.^1^ Estimates of prevalence range from 4% up to 20% in the industrial populations.^2^, ^3^, ^4^, ^5^, ^6^, ^7^ CTS is theorized to be the result of gradual swelling, causing an hourglass compression and ischemic degradation of the median nerve (MN) as it traverses the carpal tunnel.^8^ Clinical findings often, but not always, reflect the individual's degree of neural damage, which explains the variation of presenting symptoms from mild to severe pain, with/without accompanying neurological symptoms and signs.^9^ At present, a myriad of treatments exist for CTS, with non-surgical management being the first line for milder cases, and surgery reserved for those who have failed/relapsed following non-operative management.^10^, ^11^, ^12^

There is growing evidence in the literature to support the use of platelet-rich plasma (PRP) injections for those with CTS undergoing non-operative management.^13^ PRP is an autologous blood-derived biologic product consisting of concentrated platelets, which possesses many growth factors and cytokines, which are believed to reduce inflammation by augmented cellular proliferation, migration and angiogenesis.^14^^,^^15^ PRP has been shown to enhance neural tissue repair in previous in-vivo studies, resulting in Schwann cell proliferation and migration following PRP injection. A previous systematic review and meta-analysis by Catapano et al. found that PRP resulted in significant improvements patients with mild-moderate CTS.^13^ However, the included studies did not report either the results of nerve conduction studies (NCS) or the cross sectional area (CSA) of the MN in each of the included RCTs.

The purpose of this study was to perform an updated systematic review with meta-analysis on trials focusing on patient-reported outcome measures (PROMs), NCS results and CSA measurements of those who underwent PRP injection for mild-moderate CTS, versus a control. Our hypothesis was that PRP would result in moderate improvements in PROMs, NCS and CSA at short-term follow-up.

## 2 Methods

### 2.1 Search strategy

Two independent reviewers performed a systematic review of MEDLINE, EMBASE and Scopus databases in June 2020. This search was carried out based on the Preferred Reporting for Systematic Reviews and Meta-Analyses (PRISMA) guidelines. The following keywords were utilized for the search: (carpal tunnel OR carpal tunnel syndrome OR cts OR median neuropathy) AND (platelet-rich plasma OR prp). A senior author reviewed discrepancies in inclusion or exclusion of studies.

### 2.2 Inclusion and exclusion criteria

The inclusion criteria for studies included the following: 1) prospective trials examining non-operative management of CTS with PRP versus a control, 2) written in English, 3) paper published in a peer-reviewed journal, and 4) full text must have been available. The exclusion criteria for studies included the following: 1) non-randomized group used, 2) papers not published in English language, 3) papers published without peer-review, 4) retrospective studies, 5) review articles, 6) case reports, and 7) laboratory or cadaveric studies. No timeline was applied to the search to exclude studies. Using these inclusion and exclusion criteria, the titles and abstracts of each of the returned papers were screened with the full texts of potentially relevant studies subsequently reviewed. Each study's references list was then reviewed for additional articles.

### 2.3 Data analysis

Under the guidance of the senior author, data extraction from included studies was carried out independently by the same two reviewers. For each included study, the level of evidence (LOE) was assessed and evaluated based on the criteria established by *Oxford Centre of Evidence Based Medicine.*^16^ The Cochrane Collaboration risk of bias tool was used in order to evaluate risk; a study was deemed to be ‘low risk’ when every single item was scored as ‘low risk’. Studies were evaluated as moderate risk of bias when one or two items were classified as ‘high risk’ or ‘unclear risk’. Studies were deemed to be high risk if more than two items were scored as ‘high risk’.^17^ A comparison was formulated of study outcomes of CTS with PRP versus control groups.

### 2.4 Statistical analysis

Qualitative statistical analysis was performed using the *Statistical Package for the Social Sciences* (IBM Corp. Released 2013. IBM SPSS Statistics for Macintosh, Version 22.0. Armonk, NY: IBM Corp.). Meta-analysis of results was performed on the studies using *Review Manager ((RevMan) [Macintosh]. Version 5.3. Copenhagen: The Nordic Cochrane Centre, The Cochrane Collaboration, 2014.)* A p-value of <0 0.05 was deemed to be statistically significant.

## 3 Results

### 3.1 Literature search

**Fig. 1:**
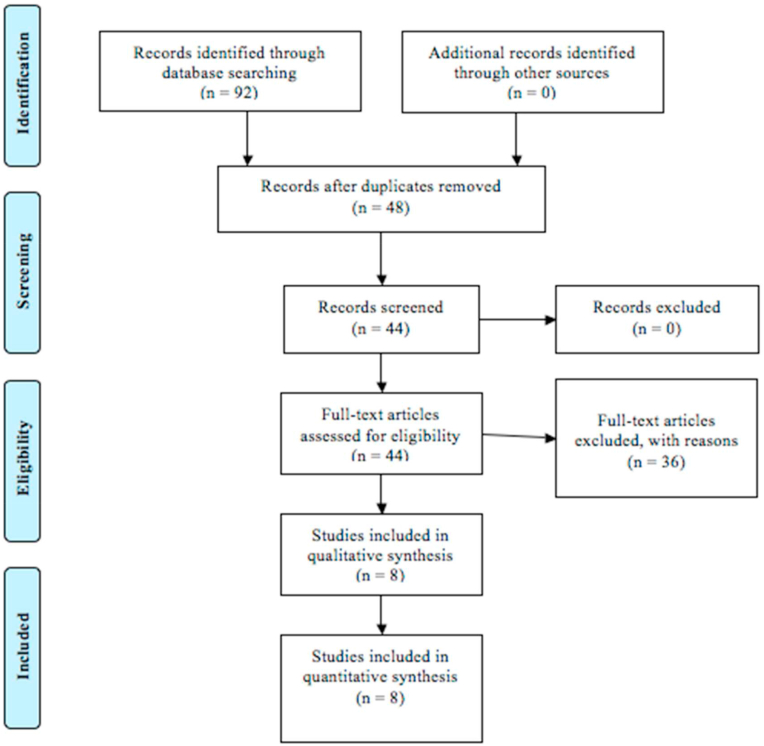
PRISMA flow chart.

The search resulted in a total of 92 studies. After removal of 48 duplicates, the abstracts of remaining articles were assessed using the inclusion and exclusion criteria. The PRISMA Flow Chart is illustrated in Fig. 1.

### 3.2 Study characteristics

A total of eight studies^18^, ^19^, ^20^, ^21^, ^22^, ^23^, ^24^, ^25^ with 404 patients (85.7% females) were included. All studies were prospective trials published focusing on the use of PRP in the non-operative management of CTS versus a control, with a mean follow-up time of 3.9 months (1.0–6.0). The patient demographics and specifics of the PRP injections are illustrated in Table 1, Table 2 respectively.

**Table 1:** Study charcteristics & patient demographics.

Author	LOE	PRP	N PRP	Female (%)	Age ± SD (Yrs)	Control	N Control	Female (%)	Age ± SD (Yrs)	F/U (Mo)	Risk of Bias
Atwa et al., 2018	II	PRP	18	16 (88.9%)	38.5 ± 8.0	CS	18	16 (88.9%)	36.6 ± 8.8	3	High
Güven et al., 2019	II	PRP & Splint	20	N/R	47.5	Splint	20	N/R	50	1	High
Malahias et al., 2018	I	PRP US	26	N/R	60.5 ± 14.4	NS	24	N/R	57.2 ± 16.1	3	Low
Raeissadat et al., 2018	I	PRP & Splint	21	21 (100%)	51.2 ± 9.8	Splint	20	20 (100%)	47.2 ± 7.1	3	High
Senna et al., 2019	I	PRP US	43	35 (81.4%)	38.3 ± 6.4	CS US	42	36 (85.7%)	40.7 ± 9.4	3	Moderate
Shen et al., 2019	I	PRP US	26	25 (96.2%)	56.8 ± 1.7	Dex	26	22 (82.6%)	58.5 ± 2.1	6	High
Uzun et al., 2017	II	PRP	20	16 (80%)	48.8 ± 5.8	CS	20	16 (80%)	48.5 ± 6.1	6	High
Wu et al., 2017	I	PRP US	30	27 (90%)	57.9 ± 1.5	Splint	30	25 (83.3%)	54.3 ± 1.3	6	High

CS; Corticosteroid, Dex; Dextrose, F/U; Follow-Up, Mo; Months, N; Number, NS; Normal Saline, PRP; Platelet Rich Plasma, SD; Standard deviation, Yrs; Years.

**Table 2:** Platelet-rich plasma injection characteristics.

Author	Preparation Kit	LR/LP	Centrifuge Time	Activating Agent
Atwa et al., 2018	N/R	N/R	3000 rpm (3 min) then 4000 rpm (15 min)	Calcium Chloride
Güven et al., 2019	N/R	N/R	100 g (15 min) then 1600 g (10 min)	N/R
Malahias et al., 2018	N/R	N/R	Double Spin (Time & rpm N/R)	N/R
Raeissadat et al., 2018	Rooyagen Kit (Arya Mabna Tashkis Corp)	LP	1600 rpm (12 min) then 3500 rpm (7 min)	Sodium Citrate & Autologous Thrombin
Senna et al., 2019	Special PRP Kit (GD Medical Pharma)	N/R	3000 rpm (3 min) then 4000 rpm (15 min)	Calcium Chloride
Shen et al., 2019	Regen Kit (Geosmatic)	LR	3400 rpm (15 min)	Sodium Citrate & Autologous Thrombin
Uzun et al., 2017	N/R	N/R	4000 rpm (10 min)	Sodium Citrate
Wu et al., 2017	Regen Kit (Geosmatic)	LR	3400 rpm (15 min)	Sodium Citrate & Autologous Thrombin

LP; Leukocyte-Poor, LR; Leukocyte-Rich, Min; Minute, N/R; Not reported, Plt Conc; Platelet Concentration, PRP; Platelet-Rich Plasma, RPM; Rounds Per Minute.

*Note: Platelet Concentration was not reported in any of the included studies.

### 3.3 Patient reported outcomes measures

**Fig. 2:**
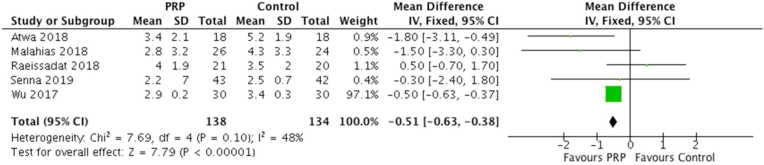
Forest Plot of VAS scores at 3 Months.

A total of five studies reported visual analogue scale (VAS) scores at three months, with 138 patients treated with PRP injection and 134 controls. For the group treated with PRP, the mean VAS score was 2.9 verses 3.5 in the control group. This difference achieved statistical significance (MD = −0.51 [0.95% CI -0.63 to −0.38]; I^2^ = 48%; P < 0.0001). The forest plot demonstrating VAS score at 3 months is illustrated in Fig. 2.

**Fig. 3:**
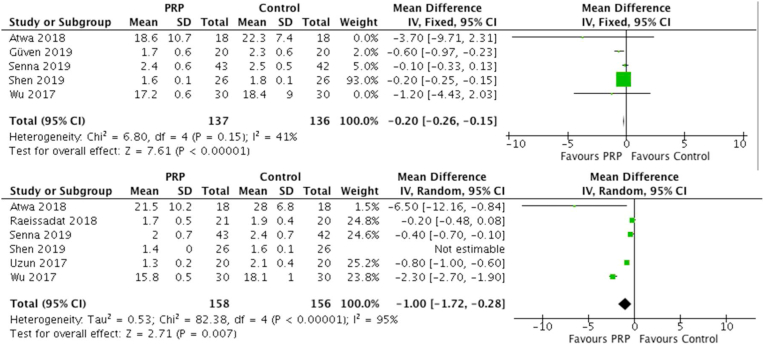
Forest Plots of SSS scores at 1 and 3 Months.

A total of five studies reported symptomatic severity scores (SSS) from the Boston carpal tunnel questionnaire (BCTQ) at 1 month, with 137 patients treated with PRP injection and 136 controls. For PRP, the mean SSS was 7.52 verses 8.46 in the control. There was a statistically significant difference between the groups (MD = −0.20 [0.95% CI -0.26 to −0.15]; I^2^ = 41%; P < 0.00001). Six studies reported SSS at 3 months, with 158 patients treated with PRP injection and 156 controls. In the PRP group, the mean SSS was 7.97 verses 8.14 in the control group. This difference was statistically significant (MD = −1.00 [0.95% CI -1.72 to −0.28]; I^2^ = 95%; P < 0.00001). The forest plot demonstrating SSS scores at 1 and 3 months are illustrated in Fig. 3.

**Fig. 4:**
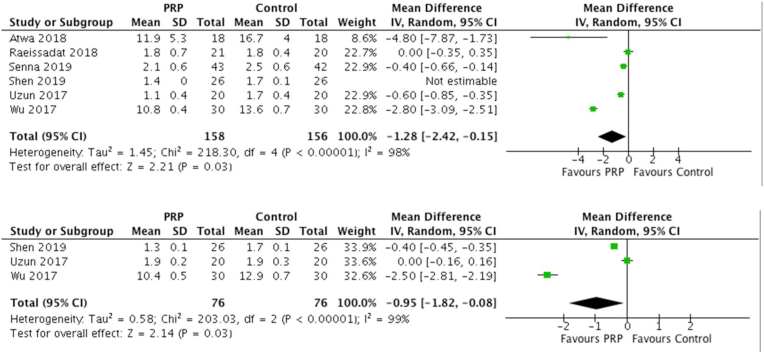
Forest Plots of FSS scores at 3 and 6 Months.

A total of six studies reported functional status scale (FSS) at three months, with 158 patients treated with PRP injection and 156 controls. For PRP, the mean FSS score was 4.59 verses 5.95 in the control group. There was a statistically significant difference between the groups (MD = −1.28 [0.95% CI -2.42 to −0.15]; I^2^ = 99%; P = 0.03). Overall, three studies reported FSS at six months, with 76 patients treated with PRP injection and 76 controls. With PRP, the mean FSS score was 5.05 verses 6.17 in the control. There was a statistically significant difference between the groups (MD = −0.95 [0.95% CI -1.81 to −0.08]; I^2^ = 99%; P = 0.03). The forest plot demonstrating FSS scores at 3 and 6 months are illustrated in Fig. 4. A summary of patient reported outcome measures is further illustrated in Table 3.

**Table 3:** Patient-reported outcome measures.

Outcome	N Studies	N PRP	PRP Mean	Control N	Control Mean	P-Value
FSS 1 Mo	5	137	5.05	136	6.17	0.57
FSS 3 Mo	6	158	4.59	156	5.95	0.03^a^
FSS 6 Mo	3	76	5.05	76	6.17	0.03^a^
SSS 1 Mo	5	137	7.52	136	8.46	<0.00001^a^
SSS 3 Mo	6	158	7.97	156	8.14	<0.00001^a^
SSS 6 Mo	3	158	6.64	156	7.58	0.23
VAS 1 Mo	3	91	2.9	90	3.4	0.51
VAS 3 Mo	5	138	2.9	134	3.5	<0.0001^a^

C; Control, FSS; Functional Status Scale, Mo; Month, N; Number, N/R; Not reported, PRP; Platelet-Rich Plasma, SSS; Symptomatic Severity, VAS; Visual Analogue Scale.

a denotes statistical significance.

### 3.4 Nerve conduction studies

A total of five studies reported distal motor latency (DML) from the NCS at 1 month, with 137 patients treated with PRP injection and 136 controls. For PRP, the mean DML was 4.8 m/s verses 4.8 m/s in the control. There was a non-statistically significant difference between the groups (MD = 0.08 [0.95% CI -0.11 to 0.27]; I^2^ = 72%; P = 0.39). Additionally, 5 studies reported DML at 3 months, with 136 patients treated with PRP injection and 136 with a control. With PRP, the mean DML was 4.7 m/s verses 4.7 m/s in the control. There was a non-statistically significant difference between the groups (MD = 0.04 [0.95% CI -0.12 to 0.20]; I^2^ = 79%; P = 0.60). Overall, 3 studies reported DML at 6 months, with 76 patients treated with PRP injection and 76 with a control. With PRP, the mean DML was 4.9 m/s verses 5.1 m/s in the control. There was a non-statistically significant difference between the groups (MD = 0.20 [0.95% CI -0.12 to 0.52]; I^2^ = 96%; P = 0.22).

A total of five studies reported sensory nerve conduction velocity (SNCV) from the NCS at 1 month, with 137 patients treated with PRP injection and 136 with a control. With PRP, the mean SNCV was 36.4 m/s verses 34.3 m/s in the control. There was a non-statistically significant difference between the groups (MD = 2.02 [0.95% CI -3.14 to 7.19]; I^2^ = 99%; P = 0.44). Additionally, 5 studies reported SNCV at 3 months, with 137 patients treated with PRP injection and 137 with a control. With PRP, the mean SNCV was 42.3 m/s verses 42.6 m/s in the control. There was a non-statistically significant difference between the groups (MD = −0.52 [0.95% CI -1.93 to 0.88]; I^2^ = 87%; P = 0.46). Overall, 3 studies reported SNCV at 6 months, with 76 patients treated with PRP injection and 76 with a control. With PRP, the mean SNCV was 33.5 m/s verses 34.3 m/s in the control. There was a non-statistically significant difference between the groups (MD = −0.85 [0.95% CI -2.44 to 0.74]; I^2^ = 89%; P = 0.30). A summary of nerve conduction studies is further illustrated in Table 4.

**Table 4:** Nerve conduction studies.

Outcome	N Studies	N PRP	PRP Mean	Control N	Control Mean	P-Value
DML (m/s) 1 Mo	5	137	4.8	136	6.17	0.39
DML (m/s) 3 Mo	5	136	4.7	136	4.7	0.60
DML (m/s) 6 Mo	3	76	4.9	76	5.1	0.22
SNCV (m/s) 1 Mo	5	137	36.4	136	34.2	0.44
SNCV (m/s) 3 Mo	5	137	42.3	137	42.6	0.46
SNCV (m/s) 6 Mo	3	76	33.5	76	34.3	0.30

C; Control, DML; Distal Motor Latency, m/s; Metres per Second, Mo; Month, N; Number, N/R; Not reported, PRP; Platelet-Rich Plasma, SNCV; Sensory Nerve Conduction Velocity.

### 3.5 Cross sectional area

**Fig. 5:**
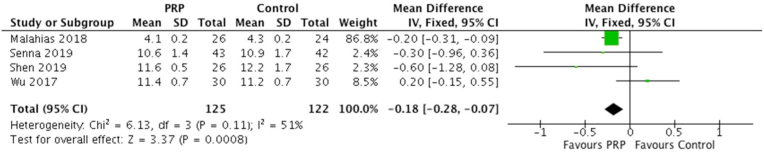
Forest plot of CSA at 3 Months.

A total of 4 studies reported CSA at 3 months, with 125 patients treated with PRP injection and 122 with a control. With PRP, the mean CSA score was 9.65 mm^2^ verses 9.95 mm^2^ in the control. There was a statistically significant difference between the groups (MD = −0.18 [0.95% CI -0.28 to −0.07]; I^2^ = 51%; P = 0.0008). The forest plot demonstrating CSA at 3 months is illustrated in Fig. 5. A summary of the cross sectional areas are further illustrated in Table 5. The supplementary appendix comprises analyses not included in included in the accompanying figures.

**Table 5:** Cross sectional area.

Outcome	N Studies	N PRP	PRP Mean	Control N	Control Mean	P-Value
CSA 1 Mo	4	119	11.85	118	11.59	0.69
CSA 3 Mo	4	125	9.65	122	9.95	0.0008^a^

C; Control, CSA; Cross Sectional Area, Mo; Month, N; Number, N/R; Not reported, PRP; Platelet-Rich Plasma.

a denotes statistical significance.

## 4 Discussion

The most important finding in this study was that, when compared to controls, the use of PRP in CTS resulted in significant improvements in both symptoms and function at short-term. While PRP resulted in significantly smaller CSA versus controls, there was no corresponding improvement in either motor or sensory results of NCS. Therefore, there is high quality evidence to validate the use of PRP in short-term symptomatic management of mild to moderate CTS, however longer term studies are required to further evaluate the longevity of these effects when compared to controls.

Pain levels were significantly lower in the short-term following PRP injection when compared to a control, with significantly lower VAS scores reported at 3 months post-intervention. However, there was only one randomized control trial (Wu et al.^25^), which reported VAS scores at six months in patients who received PRP versus a night splint control. The findings of our meta-analysis are contrary to the previous systematic review and meta-analysis performed by Catapano et al.,^13^ who that found no significant differences in VAS scores at short-term follow-up when compared to various controls. These results are promising, as the inclusion of further studies in our review has further informed the literature on this topic. Although the results of this study are predominantly positive, the relatively short-term follow-up of the included studies poses a question as to the longevity of the effect of PRP in treating patients with mild to moderate CTS. Furthermore, the fact that there is a difference in conclusion between these two recent meta-analysis (due to the increased number of trials on the topic), this highlights the current paucity of evidence in relation to the use of PRP in the non-operative management of CTS. This therefore suggests that further studies are not only necessary to validate our conclusions, but also to focus on longer-term follow-up are warranted on the topic.

First described by Levine et al.^26^ in 1993, the BCTQ has emerged as a standardized, patient-based outcome measure of symptom severity and functional status. This tool focuses on nine activities of daily living which are deemed likely to cause symptoms of MN compression in patients suffering from CTS.^27^ This meta-analysis supports previous literature that injecting PRP in the management of CTS results in significant improvements in symptomology and functionality when compared to controls.^13^^,^^28^ Previous in vivo and in vitro laboratory studies have demonstrated that PRP results in reduced inflammation of soft tissues.^29^^,^^30^ Our meta-analysis found significant improvements in both BCTQ scores (SSS and FSS) up to 6 months follow-up in those who received PRP as a non-operative management option when compared to a control. However, discrepancies in reported PRP varieties used between the included studies of this meta-analysis limit in depth evaluation of the effects of leukocyte-rich (LR) or –poor (LP) PRP at present.

Although the pathophysiology of CTS is believed to be relatively well-understood,^8^^,^^31^ the most appropriate treatment at any given point of the disease spectrum remains the subject of debate.^32^, ^33^, ^34^, ^35^ The symptoms associated with CTS are believed to occur secondary to sustained pressure causing ischemia of the MN. This ischemia progresses to demyelination and axonal loss in severe cases.^9^^,^^36^ Therefore, a larger CSA of the MN (typically measured using US or magnetic resonance imaging) is often believed to correspond to more severe symptoms as well as poorer clinical functionality.^37^, ^38^, ^39^, ^40^ The measurement of the CSA at the point of entrance of the MN at the carpal tunnel has been previously demonstrated to represent the highest sensitivity and specificity for CTS in patients with more mild or moderate disease.^37^ Our meta-analysis found that the use of PRP in patients with mild to moderate CTS resulted in smaller CSA measurements of the MN at 3 months follow-up, corresponding to significantly improved reported pain and symptomology scores. Although only two included studies reported CSA measurements at 6 months limited our ability to preform meta-analysis at this time of follow-up, Wu et al.^25^ reported significant reductions in CSA measurements using US at 6 month post-intervention in patients when compared to those used a night splint as a control.

Severe CTS has been shown to result in demyelination, deranged action potentials and ultimate axonal loss of the MN.^41^ However, previous in-vivo studies have demonstrated that the injection of PRP possesses the opportunity to enhance neural tissue repair, resulting in Schwann cell proliferation, functioning and migration following PRP injection.^42^ Despite the potential of PRP as a viable treatment option for patients with mild to moderate symptoms of CTS, our study found that no significant improvement was seen in NCS results (both DML and SNCV) in those who received PRP versus a control. However, two prospective trials previously reported significant improvements in both DML and SNCV in those who received PRP injections versus a corticosteroid (CS) control at up to 3 months follow-up.^18^^,^^22^ Despite these positive findings, numerous RCTs have failed to see significant improvements in NCS results following PRP injection in the management of mild to moderate CTS when compared to controls.^19^^,^^23^ Although the clinical findings of this study illustrate majorly promising evidence supporting the role of PRP as a non-operative treatment of CTS, limited evidence exists for its use in benefitting NCS results.^43^

### 4.1 Limitations

The authors acknowledge this study is subject to the innate limitations of being a systematic review, with the limitations in all included studies being inherent to the study as a consequence. There are many confounding factors of the included studies, none more so than the use of numerous controls including splinting alone, injection of normal saline, dextrose or corticosteroid or even no therapy, as well as the lack of standardized description of preparation and injection technique of PRP for those in the experimental arm in each of the included studies. Furthermore, Chahla et al.^44^ previously proposed that standardization of the preparation and administration of PRP may improve reporting amongst studies; this would benefit this study in reducing potential heterogeneity amongst studies include in meta-analyses similar to our study. While we initially attempted to stratify our results based on LR or LP variations of PRP, analysis was limited due to the heterogeneity. Furthermore, it would be of benefit to subgroup these further based on the number of platelets, growth factors, and other bioactive cytokines. Although these are substantial limitations in the reported PRP preparation and characteristics, the heterogeneity was low across the outcome measures, with consistent outcome measure such as VAS, SSS, FSS, DML, SNCV and CSA reported across a multitude of included studies at similar time frames of follow-up.

## 5 Conclusion

This study indicates that there may be a potential role for the use of PRP in the non-operative management of mild to moderate CTS results in improvements in pain scores, functional outcomes as well as CSA measurements of the MN at short-term follow-up. However, PRP does not result in improvements in NCS suggesting that muscle loss may occur in patients with electrophysiological evidence of deteriorating nerve function, despite PRP treatment.

## Declaration of competing interest

None.
